# Towards gender-affirming nutrition assessment: a case series of adult transgender men with distinct nutrition considerations

**DOI:** 10.1186/s12937-020-00590-4

**Published:** 2020-07-16

**Authors:** Whitney Linsenmeyer, Theresa Drallmeier, Michael Thomure

**Affiliations:** 1 Saint Louis University, Doisy College of Health Sciences, 3437 Caroline St, St. Louis, MO 63104 USA; 2 Saint Louis University, School of Medicine, 1402 South Grand Blvd, St. Louis, MO 63104 USA

**Keywords:** Transgender, Nutrition assessment, Overweight, Obesity, Diet quality, Disordered eating

## Abstract

No guidelines exist regarding nutrition assessment for transgender or gender non-conforming patients. Multiple nutrition assessment methods utilize gender-specific values that provide distinct recommendations for males and females. This clinical case series depicts the food and nutrition considerations of ten adult transgender men using anthropometric, survey, and dietary recall data. Male reference values were used to analyze patient data, though multiple approaches to nutrition assessment in the transgender population are discussed. Major nutrition-related concerns were obesity, low fruit and vegetable intake and high sodium intake; disordered eating was not a prominent concern. Further research is needed to inform nutrition care for the transgender and gender non-conforming populations.

## Background

Nutrition assessment is the first step of the Nutrition Care Process, or the systematic approach to delivering nutrition care [1]. Multiple nutrition assessment methods utilize gender-specific values in that they provide distinct recommendations for males and females. For instance, recommendations for anthropometric measures such as waist circumference and body fat percentage differ for men and women. Similarly, the Dietary Reference Intakes (DRI) reflect values for males and females.

In the United States, an estimated 0.6% of the adult population, or 1.4 million adults, identify as transgender [2]. No guidelines exist regarding nutrition assessment for transgender or gender non-conforming patients [3]. Transgender patients may opt to medically transition using hormone therapy and/or gender-affirming surgeries; these interventions may have a direct impact on nutrition related parameters such as weight status, body composition, lipid levels, blood pressure, and blood glucose levels [4–7]. A review of studies specific to transgender men identified that testosterone therapy resulted in body mass index (BMI) increases up to 11.5%, decreases in HDL-cholesterol and increases in LDL-cholesterol, and increases in hemoglobin and hematocrit [6].

The transgender population is also at an elevated risk for eating disorders. Among college students, transgender participants were four times as likely to self-report an eating disorder and over twice as likely to report compensatory behaviors such as use of diet pills, vomiting, or laxatives compared to cisgender heterosexual women [8]. Among a sample of Canadian youth and young adults, nearly half of transgender youth and more than a third of transgender young adults engaged in binge eating, fasting, pills, laxatives, or vomiting [9].

Ultimately, nutrition therapy may play an important role in mitigating the known effects of hormone therapy and promoting the overall health of transgender and gender non-conforming populations. Given the lack of nutrition recommendations for the transgender population, nutrition assessment of both clinical and psychosocial considerations is the first step towards forming a framework of gender-affirming care across the Nutrition Care Process.

## Methods

### Design

The purpose of this case series was to depict the food and nutrition considerations of adult transgender men. Ten participants were recruited using direct mail and word-of-mouth. Participants were at least 18 years of age and identified as transgender male or transmasculine, and may or may not have pursued gender-affirming medical interventions such as hormone therapy or surgeries. Transfeminine and non-binary participants were excluded from this study given that the researchers felt the nutrition considerations may be distinct enough to warrant separate studies focused on other gender identities. Participants provided written consent and were remunerated with a nominal gift card. The Saint Louis University Institutional Review Board approved this study.

### Data collection and analysis

Data collection occurred from October 2018 to February 2019 and included anthropometric, survey, and dietary recall data. The reference source for each data type is expressed in Table 1. Anthropometric data included BMI, waist circumference, and body fat percentage. Survey data included two nutrition screeners related to disordered eating: the EAT-26 and ecSI-2. The EAT-26 estimates eating disorder risk and has been validated with adolescent and adult populations; a score of 20/26 or higher was the cutoff for high eating disorder risk [10]. The ecSI-2 measures eating competence, which is characterized by a positive, comfortable, and flexible approach to food, and has been validated with various adult populations across income groups; a score of 32/48 or higher was the cutoff for high eating competence [11]. Data collection also included a patient interview; this data will be analyzed separately, but short segments were included in this case series in order to better contextualize the narrative of each case.

A diet analysis of each participant was conducted using a three-day food diary and the software ESHA Food Processor Nutrition Analysis. Energy needs to maintain weight were calculated using the Estimated Energy Requirement (EER) formula, which accounts for height, weight, age, activity level, and gender. The percentage of kcal intake from each macronutrient was compared to the Acceptable Macronutrient Distribution Ranges (AMDR) for carbohydrates, fat and protein.

Recommended Dietary Allowances (RDA) or Adequate Intake (AI) values were used for fiber, calcium, vitamin D, potassium, and iron needs. Percent intake values +/− 0–10% the Dietary Reference Intakes (DRI) were characterized as adequate; +/− 11–20% the DRI were characterized as marginally high or low; +/− > 20% the DRI were characterized as high or low.

The Dietary Guidelines for Americans (DGAs) 2015–2020 were used as the reference recommendations for saturated fat (< 10% total kcals) and sodium (< 2300 mg/day). In the case presentations, saturated fat intake of 0–10% of total kcals was characterized as appropriate; 11–15% was characterized as marginally high; > 15% was characterized as high. For sodium, intake of 0–110% the recommendation was characterized as appropriate; 111–120% was characterized as marginally high; > 120% was characterized as high.

As depicted in Table 1, many of the data types are gender-specific in that they provide specific recommendations for males and females. The research team opted to utilize the male values given that the participants were either receiving hormone therapy or intended to do so in the near future.

### Research team

An interdisciplinary clinical research team analyzed the data individually for each case and identified trends across the ten cases. The team was comprised of a registered dietitian with a research focus on nutrition care for the transgender population, a family physician who provides gender-affirming medical care for patients at a community health center, and a reproductive endocrinologist who provides gender-affirming medical care for patients at an area hospital. The research team was selected given their content expertise in medicine and dietetics, as well as their activity in both research and direct patient care with the transgender population.

### Case presentations

The following cases are presented in order of age from youngest to oldest. Coded initials are used to ensure confidentiality. The results are depicted in Table 2.

#### Case 1

**BN** is a 22 year-old transgender male and was just about to start hormone therapy. He works night shifts and notes, “I just don’t eat what I should.” BN is hopeful that his diet and exercise will improve moving forward and that “testosterone’s going to help weight distribution and things like that.”

BN has a BMI of 45 kg/m^2^, body fat percentage of 40% and waist circumference of 56 in., indicating class III obesity and central adiposity. He has a low risk of eating disorders (5/26 on the EAT-26) and yet a low degree of eating competence (16/48 on the ecSI-2). His dietary pattern includes 3–4 eating instances per day at home, work, or fast food restaurants. BN consumes 0–1 servings of fruits and vegetables per day and has a high intake of added sugars in the form of sodas, sweetened tea, and ice cream.

BN’s diet analysis was characterized by low energy (59% kcal needs), appropriate saturated fat (10% kcal intake), low fiber (45% AI) and high sodium (149% DGAs limit). His macronutrient intake was 48% carbohydrate, 37% fat and 15% protein, or within recommended AMDR ranges. BN’s micronutrient intake reflect low calcium (40% RDA), vitamin D (3% RDA), potassium (36% AI) and marginally high iron intake (114% RDA).

#### Case 2

**CE** is a 27 year-old transgender male. He gained 40 lbs. when starting hormone therapy 18 months ago. CE reported that he “tried really hard for like, about 6-8 months to lose it, and then I was getting nowhere. So, I just kind of gave up.” He also described himself as a picky eater and “weird about textures,” but that he is starting to try new foods that his boyfriend prepares.

He has a BMI of 33 kg/m^2^, body fat percentage of 34% and waist circumference of 41 in., indicating obesity class I and central adiposity. He has a low risk of eating disorders (6/26 on the EAT-26) and yet a low degree of eating competence (24/48 on the ecSI-2). CE’s dietary pattern includes 2–4 eating instances per day at home and work. He consumes 0–1 servings of vegetables per day and a high intake of added sugars in the form of sodas, iced coffees and energy drinks.

His diet analysis was characterized by low energy (74% kcal needs), appropriate saturated fat intake (10% kcal intake), low fiber (41% AI), and high sodium (206% DGAs limit). His macronutrient intake was 63% carbohydrate, 23% fat and 14% protein, or within recommended AMDR ranges. CE’s micronutrient intake reflected adequate calcium (105% RDA), low vitamin D (48% RDA), low potassium (40% AI), and marginally low iron (84% RDA).

#### Case 3

**VK** is a 29 year-old transgender male and has been on hormone therapy for 5 years. He noted that although he has “always struggled with food and weight and body image,” that his relationship with food and exercise started improving when he came out. He now approaches food as “more of a tool and a strengthening thing.”

VK has a BMI of 35 kg/m^2^, body fat percentage of 35% and waist circumference of 40 in., indicating obesity class II and central adiposity. He has a low risk of eating disorders (16/26 on the EAT-26) and yet a low degree of eating competence (30/48 on the ecSI-2). His dietary pattern includes 4–5 eating instances per day at home and fast food restaurants. VK consumes 2–3 servings of fruits and vegetables daily and added sugars in the form of sweetened coffee drinks and energy bars.

VK’s diet analysis was characterized by low energy (58% kcal needs), high saturated fat (29% kcal intake), low fiber (32% AI), and marginally high sodium (117% DGAs limit). His macronutrient intake was 21% carbohydrate, 55% fat and 24% protein, or lower in carbohydrate and higher in fat than the AMDR ranges. VK’s micronutrient intake reflected high calcium (124% RDA), low vitamin D (7% RDA), low potassium (16% AI) and adequate iron (102% RDA).

#### Case 4

**MR** is a 30 year-old transgender male and has been on hormone therapy for just over 1 year. He reported gaining 15 lbs. of mostly muscle mass during the first 2 months of hormone therapy and then losing weight after a reconstructive chest surgery, or top surgery. He notes, “I don’t necessarily want to be thinner.” MR is somewhat conscientious of his kcal intake but is primarily driven by the feeling: “I still want to have a lot of fun, and I want to be myself.”

He has a BMI of 25 kg/m^2^, body fat percentage of 19% and waist circumference of 32 in., indicating an overweight BMI and yet overall healthy body weight and composition given the additional anthropometric measures. He has a low risk of eating disorders (3/26 on the EAT-26) and yet a low degree of eating competence (29/48 on the ecSI-2). MR’s follows a mostly vegetarian dietary pattern with 3–4 eating instances per day at home and at restaurants. He consumes 2–3 servings of vegetables and fruits daily, very little added sugars, and 1–4 alcoholic drinks/day.

MR’s diet analysis was characterized by marginally low energy (84% kcal needs), appropriate saturated fat (8% total kcals), low fiber (71% AI), and high sodium (134% DGAs limit). His macronutrient intake was 32% carbohydrate, 33% fat, and 22% protein, or lower in carbohydrate than the AMDR ranges. Additionally, 13% of his kcals were derived from alcohol. MR’s micronutrient intake reflected high calcium (132% RDA), low vitamin D (53% RDA), low potassium (47% AI) and high iron intake (171% RDA).

#### Case 5

**CA** is a 31 year-old transgender male. He started hormone therapy and had top surgery over 3 years ago. CA was motivated to lose weight in order to be eligible for top surgery, but didn’t feel comfortable going to the gym without wearing a binder, or a compression undershirt worn to flatten breasts, which he knew was not safe. CA also had gastric sleeve surgery after his top surgery “just because I wanted to take care of my health more.” He notes, “I feel like I made the right choice in doing top surgery first, and then that lead me to take care of myself more.”

CA has a BMI of 33 kg/m^2^, body fat percentage of 41% and waist circumference of 44 in., indicating class I obesity and central adiposity. He has a low risk of eating disorders (3/26 on the EAT-26) and yet a low degree of eating competence (23/48 on the ecSI-2). He follows a vegetarian dietary pattern with 5–6 eating instances per day at home and fast food restaurants. CA consumes 2–3 servings of fruits and vegetables daily and added sugars in the form of breakfast cereals, muffins, and sweetened tea.

CA’s diet analysis was characterized by low energy (77% kcal needs), marginally high saturated fat (13% total kcals), low fiber (69% AI) and high sodium (154% DGAs limit). His macronutrient intake was 47% carbohydrate, 37% fat and 15% protein, or slightly higher in fat than the AMDR ranges. CA’s micronutrient intake reflected adequate calcium (90% RDA), low vitamin D (11% RDA), low potassium (15% AI) and high iron (199% RDA).

#### Case 6

**GT** is a 32 year-old transgender male. He has been on hormone therapy for over 10 years and recalls initially gaining approximately 40 lbs., which he lost slowly over time. GT and his partner are now into ayurvedic cooking, though he still enjoys “going to get a burger across the street.” GT is physically active with drumming, biking and rock climbing.

He has a BMI of 25 kg/m^2^, body fat percentage of 15% and waist circumference of 35 in., indicating an overweight BMI and yet overall healthy body weight and composition given the additional anthropometric measures. He has a low risk of eating disorders (2/26 on the EAT-26) and yet a low degree of eating competence (30/48 on the ecSI-2). GT’s dietary pattern includes 5–7 eating instances per day at home, a friend’s home, or restaurants. He consumes 3–6 servings of fruits and vegetables daily and sugar-sweetened foods and beverages in the form of coffee drinks, cookies and candy. His beverage intake also includes 0–4 alcoholic drinks per day.

GT’s diet analysis was characterized by marginally high energy (119% kcal needs), marginally high saturated fat (14% total kcals), low fiber (59% AI) and high sodium (221% DGAs limit). His macronutrient intake was 43% carbohydrate, 36% fat and 16% protein, or slightly lower in carbohydrate and higher in fat than the AMDR ranges. Additionally, 5% of his kcals were derived from alcohol. GT’s micronutrient intake reflected adequate calcium (98% RDA), low vitamin D (3% RDA), marginally low potassium (89% DI) and high iron (263% RDA).

#### Case 7

**CM** is a 33 year-old transgender male and has been on hormone therapy for over 5 years. He experienced an initial weight gain of approximately 60 lbs., both due to an increase in appetite and a genuine desire to have a larger body size. He describes this as, “I’m not going to blow over and am just more rugged, solid, stocky.”

CM has a BMI of 51 kg/m^2^, body fat percentage of 46% and waist circumference of 57 in., indicating class III obesity and central adiposity. He has a low risk of eating disorders (12/26 on the EAT-26) and a high degree of eating competence (35/48 on the ecSI-2). CM’s dietary pattern includes 5–6 eating instances per day at home, work, or fast food restaurants. He consumes 1–2 servings of vegetables per day and added sugars in the form of candy, energy drinks, and ice cream. His beverage intake also includes 0–2 alcoholic drinks per day.

CM’s diet analysis was characterized by adequate energy (102% kcal needs), marginally high saturated fat (14% total kcals), low fiber (71% AI) and high sodium (228% DGAs limit). His macronutrient intake was 42% carbohydrate, 41% fat and 15% protein, or lower in carbohydrate and higher in fat than the AMDR ranges. Additionally, 2% of his kcals were derived from alcohol. CM’s micronutrient intake reflected high calcium (167% RDA), low vitamin D (11% RDA), low potassium (37% AI) and high iron (149% RDA).

#### Case 8

**SB** is a 37 year-old transgender male and has been on hormone therapy for just over 1 year. He was highly motivated to improve his diet prior to starting hormone therapy. He noted, “… the things I was able to eat, in my mind, were helping me to get ready for the medical transition.” He had also sought out foods that had been purported to increase natural levels of testosterone, though he noted he doesn’t know if they actually worked or simply helped him to feel more mentally prepared.

SB has a BMI of 21 kg/m^2^, body fat percentage of 13% and waist circumference of 31 in., indicating a healthy body weight and body composition. He has a low risk of eating disorders (7/26 on the EAT-26) and a high degree of eating competence (48/48 on the ecSI-2). SB follows a gluten-free dietary pattern with 4–7 eating instances per day at home or work. He consumes 2–4 servings of fruits and vegetables per day and minimal added sugars in any form.

SB’s diet analysis was characterized by adequate energy (109% kcal needs), appropriate saturated fat (7% total kcals), low fiber (75% AI) and high sodium (169% DGAs limit). His macronutrient intake was 44% carbohydrate, 38% fat and 18% protein, or slightly lower in carbohydrate and higher in fat than the AMDR ranges. SB’s micronutrient intake reflected low calcium (53% RDA), low vitamin D (20% RDA), low potassium (39% AI) and high iron (197% RDA).

#### Case 9

**CJ** is a 49 year-old transgender male and has been on hormone therapy for over 11 years. CJ’s diet or physical activity level has not changed over the years in relation to his transition. He comments, “I don’t think transitioning has changed anything about the way I eat, really.” Rather, his diet fluctuates more so in relation to his income and employment benefits.

CJ has a BMI of 31 kg/m^2^, body fat percentage of 30% and waist circumference of 42 in., indicating obesity class I and central adiposity. He has a low risk of eating disorders (2/26 on the EAT-26) and yet a low degree of eating competence (26/48 on the EAT-26). CJ’s dietary pattern includes 3–4 eating instances per day at home, restaurants, or in his car. He consumes 0–3 servings of fruits and vegetables per day and added sugars in the form of candy, pastries, cookies, and coffee drinks. His beverage intake also includes low carbohydrate energy drinks.

CJ’s diet analysis was characterized by marginally high energy (112% kcal needs), high saturated fat (18% total kcals), low fiber (49% AI) and high sodium (205% DGAs limit). His macronutrient intake was 38% carbohydrate, 50% fat and 12% protein, or lower in carbohydrate and higher in fat than the AMDR ranges. CJ’s micronutrient intake reflected marginally high calcium (111% RDA), low vitamin D (3% RDA), low potassium (18% AI) and high iron (158% RDA).

#### Case 10

**GJ** is a 51 year-old transgender male and has been on hormone therapy for 7 years. His diet has not changed much throughout his transition. He notes, “It’s pretty much been the same throughout.” GJ points out that much of the LGBT social community is centered around bars and that “you end up having a lot of calories of alcohol … if you center around a bar, you know, your eating habits and drinking habits are not the greatest.”

He has a BMI of 39 kg/m^2^, body fat percentage of 34% and waist circumference of 44 in., indicating obesity class II and central adiposity. He has a low risk of eating disorders (8/26 on the EAT-26) and yet a low degree of eating competence (23/48 on the EAT-26). GJ’s dietary pattern includes 3–4 eating instances per day. He consumes 0–2 servings of vegetables per day and sugar-sweetened foods and beverages in the form of soda and cookies. His beverage intake also includes 0–3 alcoholic drinks per day.

GJ’s diet analysis was characterized by adequate energy (109% kcal needs), appropriate saturated fat (7% total kcals), low fiber (39% AI) and high sodium (155% DGAs limit). GJ’s macronutrient intake was 64% carbohydrate, 24% fat and 8% protein, or slightly lower in protein than the AMDR ranges. Additionally, 4% of his kcals were derived from alcohol. GJ’s micronutrient intake reflected low calcium (22% RDA), low vitamin D (0% RDA), low potassium (9% AI) and low iron (70% RDA).

## Discussion

### Body weight and composition

Across the 10 patients presented in this case series, 7 (70%) were obese according to the Center for Disease Control (CDC) BMI standards. Three (30%) were obese class I, 2 (20%) were obese class II, and 2 (20%) were obese class III [12]. Based on national survey data, 65.9% of transgender men are overweight or obese, and transgender males are the most likely to be obese across the lesbian, gay, bisexual, and transgender (LGBT) subgroups (46%) [13]. The findings of these case series reinforce existing assertions of obesity as a public health concern among the LGBT community and elevates the importance for transgender men in particular.

Weight gain and changes in fat distribution are known effects of both masculinizing and feminizing hormone therapy. In a 2017 meta-analysis, transgender men experienced a net average weight gain of 3.7 lbs., with a loss in body fat and a gain in lean body mass [14]. However, multiple participants in this case series reported much greater weight gain; both TG and CE reported a 40 lb. weight gain, and CM gained 60 lbs. after starting hormone therapy. While hormone therapy may directly result in modest weight gain, overweight and obesity are multifactorial conditions influenced by diet, lifestyle, and genetic factors. The transgender community may be particularly vulnerable due to minority stressors and barriers to healthcare. It is also notable that patients may desire a larger body size given their ideal masculine or feminine body type, as was the case with CM. Further research is needed to explore weight management strategies that are both effective and culturally accepted by the transgender community.

Although two patients in this case series (MR and TG) were overweight by CDC standards, their body fat percentage and waist circumferences were within healthy ranges. This suggests a higher degree of lean body mass and therefore a mislabeling of overweight. Existing population-level data on the transgender population utilizes BMI and does not include waist circumference or body composition analyses for inherent logistical reasons. The findings of this case series suggests that some transgender men may be mislabeled as overweight, which is an inherent limitation of BMI among all populations. In addition to the foundational work by Klaver et al., this is one of the first studies to include measures of body weight, body fat percentage, and waist circumference to provide a more accurate description of weight status [14, 15].

### Diet analysis

Participants reported a range of kcal intake from 58 to 110% of their estimated needs. Variations may be due to underreporting or alterations in food intake knowing the data would be analyzed, or an intentional kcal deficit to support weight loss.

None of the ten participants met the federal recommendations for fiber or fruit and vegetable intake, all ten reported a diet high or marginally high in sodium, and half reported a diet high or marginally high in saturated fat. The average fiber intake among adults in the United States is 17 g per day; 87.1% of the population does not meet the recommendations for daily fiber intake [16]. Nationally, 9.2% of men meet the federal fruit intake recommendations and 7.6% meet the federal vegetable intake recommendations [17]. An estimated 89% of adults consume excess sodium and 65% consume excess saturated fat [18, 19]. Thus, the trends among participants in this study are consistent with the existing estimates that most adults do not meet the federal recommendations for fiber, fruit and vegetable intake, sodium, or saturated fat.

Regarding micronutrient intake, all participants reported diets low or marginally low in potassium, which is likely second to the low intake of fruits and vegetables. Several participants reported diets high in calcium and iron, though the reported values did not approach the Tolerable Upper Intake Levels (UL) for these minerals. Lastly, all participants reported diets low in vitamin D, though a more accurate assessment of vitamin D would account for endogenous synthesis from sun exposure. In comparison, 91.5% of adult men in the United States are at risk for inadequate vitamin D intake from foods alone, 26.0% for inadequate calcium intake, 35.0% for potassium intake, and 0.2% for inadequate iron intake [20].

Regarding fast food, seven participants reported consuming fast food at least once during the 3 day reporting period. In comparison, 37% of U.S. adults consume fast food on a given day [21]. Regarding alcohol, four participants in this case series reported alcohol consumption on at least one of the 3 days. Though some exceeded the moderate alcohol intake recommendations, no participants quite met the threshold for binge drinking of five or more drinks on the same occasion [19]. In comparison, an average of 11.1% of men in the United States have a mean alcohol intake that exceeds recommendations and 71.3% of men exceed the maximum recommendation for number of drinks on a given day [22].

### Gender-specific values

As depicted in Table 1, multiple nutrition assessment methods are gender-specific, meaning the reference source indicates separate values for males and females. Within the anthropometric data collected, waist circumference and body fat percentage values were gender-specific. Within the diet analysis, total energy, fiber, calcium, vitamin D, potassium, and iron levels were gender-specific. This raises the logical question of how to approach nutrition assessment for transgender and gender non-conforming patients. No standards of care exist regarding nutrition assessment methods. Potential approaches are discussed below.

#### Use values consistent with the patient’s gender identity

Clinicians may opt to utilize values consistent with the patient’s gender identity. In this case series, we used the reference values for males across all data types. This may be a step towards gender-affirming nutrition assessment and may build rapport with patients. However, this approach may potentially misrepresent a patient’s nutrition status. For example, a patient with a waist circumference of 37 in. would be characterized as high for a female (> 35 in.) but acceptable for a male (< 40 in.).

#### Individualize assessment to align with the patient’s medical transition

Another approach may be to individualize nutrition assessment to align as closely as possible with the patient’s medical transition, especially regarding the duration of hormone therapy. This case series depicted a range of patient histories with regards to the initiation of hormone therapy. CJ had been on hormone therapy for over 11 years, and BN was starting hormone therapy the very next day.

The World Professional Association for Transgender Health outlines the effects, expected onset, and expected maximum effect of both masculinizing and feminizing hormones with regards to body fat redistribution and changes in muscle mass. For example, an increase in muscle mass and strength has an expected onset of 6–12 months and an expected maximum effect of 2–5 years in patients using masculinizing hormones [7]. Clinicians may switch from the female to male values for body fat assessment once a patient has been on masculinizing hormones for 12 months. However, clinicians should note that not all patients that identify as transgender will use hormone therapy or have gender-affirming surgeries.

#### Express data as a range between male and female values

In certain aspects of nutrition assessment, it may be realistic to express data as a range between the female and male values. For example, using the Estimated Energy Requirement (EER) equation, the estimated energy needs for a 30 year-old, 5 ft, 8 in. tall patient weighing 140 lbs. and classified as “low active” are: 2366 kcals/day for a female and 2534 kcals/day for a male. Therefore, energy needs for a transgender female or transgender male patient of the same height, weight, and activity level may differ by only a few hundred kcals and can be expressed as a range of 2366–2534 kcals/day.

### Disordered eating

None of the patients in this case series screened positive for disordered eating using the EAT-26 [10]. Existing research suggests that the transgender community is at an increased risk for disordered eating and eating disorders [8]. Possible explanations were that this small sample was simply not representative of the transgender population, did not include transgender females, or that the measures used in this study were not comparable to the measures used in the existing research that has focused on LGBT youth and transgender women [8, 9, 23, 24]. Given the sampling strategy, it is also possible that potential participants declined to participate knowing their food intake would be scrutinized. Future research is needed to explore eating disorder prevalence within the transgender population with attention to subgroups based on gender identity, age, race, education level, and socioeconomic status.

Only two of the patients scored a high degree of eating competence using the ecSI-2.0 [11]. Although we expected a negative screen on the EAT-26 to align with a high degree of eating competence on the ecSI-2.0, this was not the case. This may be due to the fact that the two measures are designed to assess two different qualities— risk of an eating disorder versus a positive, comfortable, and flexible approach to food— and that the nature of one’s eating pattern can span a broad spectrum of mindsets and behaviors. While the extant literature has centered on eating disorder prevalence, future studies may explore the related yet distinct concept of eating competence within the transgender population.

### Future research

Future research is needed to explore other aspects of nutrition assessment, such as laboratory values and a culturally appropriate nutrition-focused physical exam. Given that this case series focused exclusively on transgender males, further research is needed to understand the distinct nutrition-related considerations for transgender female and gender non-conforming populations.

## Conclusions

Many aspects of nutrition assessment depend on male or female reference values. No standards of care exist to inform clinicians on nutrition assessment methods for transgender and gender non-conforming patients. The ten cases here represent narratives of transgender men with distinct nutrition considerations. Clinicians may opt to use the values related to a patient’s gender identity, may individualize nutrition care based on the patient’s stage of medical transition, or may utilize a range of values where appropriate. Further research is critical to informing a gender-affirming approach to the Nutrition Care Process.

## Supplementary information

**Additional file 1: Table 1.** Gender-specific nature of nutrition-related anthropometric, survey, and diet analysis reference sources.

**Additional file 2: Table 2.** Anthropometric, disordered eating, and diet analysis data of ten transgender males.

## Data Availability

All data generated or analyzed during this study are included in this published article.

## Abbreviations

AMDR: Acceptable macronutrient distribution range
AI: Adequate intake
BMI: Body mass index
CDC: Center for Disease Control and Prevention
DGAs: Dietary Guidelines for Americans
DRI: Dietary reference intakes
EER: Estimated energy requirement
LGBT: Lesbian, gay, bisexual, and transgender
RDA: Recommended dietary allowance
UL: Tolerable upper intake levels

## References

[1] Swan WI, Vivanti A, Hakel-Smith NA (2017). Nutrition care process and model update: towards realizing people-centered care and outcomes management. J Acad Nutr Diet.

[2] Flores AR, Herman JL, Gates GJ, Brown TN. UCLA Law, The Williams Institute. How many adults identify as transgender in the United States?https://williamsinstitute.law.ucla.edu/wp-content/uploads/How-Many-Adults-Identify-as-Transgender-in-the-United-States.pdf. Published June 2016. Accessed 15 Oct 2019.

[3] Rahman R, Linsenmeyer WR (2019). Caring for transgender patients and clients: nutrition-related clinical and psychosocial considerations. J Acad Nutr Diet.

[4] Deutsch MB, Bhakri V, Kubicek K (2015). Effects of cross-sex hormone treatment on transgender men and women. Obstet Gynecol.

[5] Fernandez JD, Tannock LR (2016). Metabolic effects of hormone therapy in transgender patients. Endocr Pract.

[6] Vehlo I, Fighera TM, Ziegelmann PK, Spritzer PM (2017). Effects of testosterone therapy on BMI, blood pressure, and laboratory profile of transgender men: a systematic review. Andrology.

[7] Coleman E, Bockting W, Botzer M (2012). World professional Association for Transgender Health. Standards of Care for the Health of transsexual, transgender, and gender-nonconforming people, version 7. Int J Trandgenderism.

[8] Diemer EW, Grant JD, Munn-Chernoff MA, Patterson DA, Duncan AE (2015). Gender identity, sexual orientation, and eating-related pathology in a national sample of college students. J Adolesc Health.

[9] Watson RJ, Veale JF, Saewyc EM (2017). Disordered eating behaviors among transgender youth: probability profiles from risk and protective factors. Int J Eat Disord.

[10] Garnet DM, Olmsted MP, Bohy Y, Garfinkel PE (1982). The eating attitudes test: psychometric features and clinical correlates. Psychol Med.

[11] Godleski S, Lohse B, Krall JS (2019). Satter eating competence inventory subscale restructure after confirmatory factor analysis. J Nutr Educ Behav.

[12] Center for Disease Control and Prevention. Adult Body Mass Index (BMI). https://www.cdc.gov/obesity/adult/defining.html. Reviewed April 11, 2017. Accessed October 15, 2019.

[13] Warren JC, Smalley KB, Barefoot KN (2016). Differences in psychosocial predictors of obesity among LGBT subgroups. LGBT Health.

[14] Klaver M, Dekker MJHJ, de Mutsert R, Twisk JWR, den Heijer M (2017). Cross-sex hormone therapy in transgender persons affects total body weight, body fat and lean body mass: a meta-analysis. Andrologia.

[15] Klaver M, De Blok CJM, Wiepjes CM (2018). Changes in regional body fat, lean body mass and lean body shape in trans persons using cross-sex hormonal therapy: results from a multicenter prospective study. Eur J Endocrinol.

[16] Han S, Wu L, Wang W, Li N, Wu X (2019). Trends in dietary nutrients by demographic characteristics and BMI among US adults, 2003-2016. Nutrients.

[17] Lee-Kwan SH, Moore LV, Blanck HM, Harris DM, Galuska D (2017). Disparities in state-specific adult fruit and vegetable consumption—United States, 2015. MMWR Morb Mortal Wkly Rep.

[18] Jackson SL, Coleman King SM, Zhao L, Cogswell ME (2016). Prevalence of excess sodium intake in the United States—NHANES, 2009–2012. MMWR Morb Mortal Wkly Rep.

[19] U.S. Department of Health and Human Services and U.S. Department of Agriculture. 2015–2020 Dietary Guidelines for Americans, 8^th^ edition. December 2015. Available at https://health.gov/dietaryguidelines/2015/resources/2015-2020_Dietary_Guidelines.pdf.

[20] Cowan AE, Jun S, Tooze JA, Eicher-Miller HA (2020). Total usual micronutrient intakes compared to the dietary reference intakes among U.S. adults by food security status. Nutrients.

[21] National Center for Health Statistics. NCHS Fact Sheet. January 2019. Available at https://www.cdc.gov/nchs/data/factsheets/factsheet_nutrition.pdf.

[22] Ricci C, Schutte AE, Schutte R, Smuts CM, Pieters M. Trends in alcohol consumption in relation to cause-specific and all-cause mortality in the United States: a report from the NHANES linked to the US mortality registry. Am J Clin Nutr 2020;111(3):580–589. https://doi.org/10.1093/ajcn/nqaa008. 10.1093/ajcn/nqaa008 31978218

[23] Donaldson AA, Hall A, Neukirch J (2018). Multidisciplinary care considerations for gender nonconforming adolescents with eating disorders: a case series. Int J Eat Disord.

[24] Gordon AR, Austin SB, Krieger N, White Hughto JM, Reisner SL. “I have to constantly prove to myself, to people, that I fit the bill”: perspectives on weight and shape control behaviors among low-income, ethnically diverse young transgender women. Soc Sci Med 2016;165:141–149. https://doi.org/10.1016/j.socscimed.2016.07.038. 10.1016/j.socscimed.2016.07.038 PMC5241136 27518756

