# Supplementary material for: Towards gender-affirming nutrition assessment: a case series of adult transgender men with distinct nutrition considerations
Source: Nutr J. 2020 Jul 16;19:74. doi: 10.1186/s12937-020-00590-4 (PMC7367386; doi:10.1186/s12937-020-00590-4)
Supplement: Supplementary file 1 — Additional file 1: Table 1. Gender-specific nature of nutrition-related anthropometric, survey, and diet analysis reference sources. [file 12937_2020_590_MOESM1_ESM.docx]

Table 1. Gender-specific nature of nutrition-related anthropometric, survey, and diet analysis reference sources.

| **Data Type** | **Reference Source** | **Gender-specific**^1^ |
| --- | --- | --- |
| ***Anthropometric*** | | |
| Body Mass Index | Center for Disease Control and Prevention | No |
| Waist circumference | American Heart Association,  National Heart, Lunch and Blood Institute | Yes |
| Body fat percentage | Academy of Nutrition and Dietetics,  National Research Council | Yes |
| ***Survey*** | | |
| EAT-26 | See references | No |
| ecSI-2 | See references | No |
| ***Diet Analysis/Nutrients:*** | | |
| Energy/kcals | DRI^2^ (Estimated Energy Requirement^3^) | Yes |
| Carbohydrate | DRI^2^ (Acceptable Macronutrient Distribution Range^4^) | No |
| Fat | DRI^2^ (Acceptable Macronutrient Distribution Range^4^) | No |
| Protein | DRI^2^ (Acceptable Macronutrient Distribution Range^4^) | No |
| Saturated fat | DGAs^5^: <10% total kcals | No |
| Sodium | DGAs^5^: <2,300 mg/day | No |
| Fiber | DRI^2^ (Adequate Intake) | Yes |
| Calcium | DRI^2^ (Recommended Dietary Allowance^6^) | Yes |
| Vitamin D | DRI^2^ (Recommended Dietary Allowance^6^) | Yes |
| Potassium | DRI^2^ (Adequate Intake^7^) | Yes |
| Iron | DRI^2^ (Recommended Dietary Allowance^6^) | Yes |

^1^Gender-specific: reference source has separate values for males and females.

^2^Dietary Reference Intakes: umbrella term regarding goals for nutrient intake.

^3^Estimated Energy Requirement: energy needs to maintain weight.

^4^Acceptable Macronutrient Distribution Range: recommended range of macronutrient intake as a percentage of total kcals—carbohydrates 45-65%, fat 20-35%, protein 10-35%.

^5^Dietary Guidelines for Americans 2015-2020: national guidelines designed to promote health and prevent disease.

^6^Recommended Dietary Allowances: average daily amount of a nutrient considered adequate to meet the needs of most healthy people.

^7^Adequate Intake: goal for nutrient intake when an RDA cannot be set.
