# Supplementary material for: Towards gender-affirming nutrition assessment: a case series of adult transgender men with distinct nutrition considerations
Source: Nutr J. 2020 Jul 16;19:74. doi: 10.1186/s12937-020-00590-4 (PMC7367386; doi:10.1186/s12937-020-00590-4)
Supplement: Supplementary file 2 — Additional file 2: Table 2. Anthropometric, disordered eating, and diet analysis data of ten transgender males. [file 12937_2020_590_MOESM2_ESM.docx]

|  | **BN** | **CE** | **VK** | **MR** | **CA** | **GT** | **CM** | **SB** | **CJ** | **GJ** |
| --- | --- | --- | --- | --- | --- | --- | --- | --- | --- | --- |
| **Anthropometrics** | | | | | | | | | | |
| BMI | 45 | 33 | 35 | 25 | 33 | 25 | 51 | 21 | 31 | 39 |
| BMI Classification | Obese III | Obese I | Obese II | Overweight | Obese I | Overweight | Obese III | Normal Weight | Obese I | Obese II |
| Body Fat % | 40% | 34% | 35% | 19% | 41% | 15% | 46% | 13% | 30% | 34% |
| Body Fat Classification | Obese | Obese | Obese | Average | Obese | Athletic | Obese | Athletic | Obese | Obese |
| Waist Circumference | 56” | 41” | 40” | 32” | 44” | 35” | 57” | 31” | 42” | 44” |
| Waist Circumference Classification | High | High | High | Normal | High | Normal | High | Normal | High | High |
| **Disordered Eating Screeners** | | | | | | | | | | |
| EAT-26 Score (Interpretation) | 5 (Low) | 6 (Low) | 16 (Low) | 3 (Low) | 3 (Low) | 2 (Low) | 12 (Low) | 7 (Low) | 2 (Low) | 8 (Low) |
| ecSI-2 Score (Interpretation) | 16 (Low) | 24 (Low) | 30 (Low) | 29 (Low) | 23 (Low) | 30 (Low) | 35 (High) | 48 (High) | 26 (Low) | 23 (Low) |
| **Diet Analysis** | | | | | | | | | | |
| Energy Intake (% EER) | 59% | 74% | 58% | 84% | 77% | 119% | 102% | 109% | 112% | 109% |
| Sat. Fat Intake (% total kcals) | 10% | 10% | 29% | 8% | 13% | 14% | 14% | 7% | 18% | 7% |
| Sodium Intake (% DGAs limit) | 149% | 206% | 117% | 134% | 154% | 221% | 228% | 169% | 205% | 155% |
| Fiber Intake (% AI) | 45% | 41% | 32% | 71% | 69% | 59% | 71% | 75% | 49% | 39% |
| Calcium Intake (% RDA) | 39% | 105% | 124% | 132% | 90% | 98% | 167% | 53% | 111% | 22% |
| Vitamin D Intake (% RDA) | 3% | 50% | 7% | 53% | 11% | 3% | 11% | 21% | 3% | 0% |
| Potassium Intake (% AI) | 36% | 40% | 16% | 47% | 15% | 89% | 37% | 39% | 18% | 9% |
| Iron Intake (% RDA) | 134% | 84% | 102% | 171% | 119% | 269% | 149% | 197% | 158% | 70% |
| Carbohydrate (grams) | 280 | 318 | 77 | 162 | 251 | 321 | 353 | 282 | 267 | 493 |
| Fat (grams) | 82 | 52 | 93 | 74 | 88 | 121 | 156 | 110 | 155 | 80 |
| Protein (grams) | 66 | 68 | 90 | 108 | 81 | 120 | 131 | 117 | 86 | 61 |
| Alcohol (grams) | 0 | 0 | 0 | 38 | 0 | 19 | 7 | 0 | 0 | 19 |

Table 2. Anthropometric, disordered eating, and diet analysis data of ten transgender males.
